# Does plantar skin abrasion affect cutaneous mechanosensation?

**DOI:** 10.14814/phy2.15479

**Published:** 2022-10-18

**Authors:** Bert Wynands, Claudio Zippenfennig, Nicholas B. Holowka, Daniel E. Lieberman, Thomas L. Milani

**Affiliations:** ^1^ Department of Human Locomotion, Institute of Human Movement Science and Health Chemnitz University of Technology Chemnitz Germany; ^2^ Department of Anthropology, College of Arts and Sciences University at Buffalo Buffalo New York USA; ^3^ Department of Human Evolutionary Biology Harvard University Cambridge Massachusetts USA

**Keywords:** callus, cutaneous mechanoreceptors, monofilaments, plantar sensitivity, skin properties, vibration thresholds

## Abstract

In humans, plantar cutaneous mechanoreceptors provide critical input signals for postural control during walking and running. Because these receptors are located within the dermis, the mechanical properties of the overlying epidermis likely affect the transmission of external stimuli. Epidermal layers are highly adaptable and can form hard and thick protective calluses, but their effects on plantar sensitivity are currently disputed. Some research has shown no effect of epidermal properties on sensitivity to vibrations, whereas other research suggests that vibration and touch sensitivity diminishes with a thicker and harder epidermis. To address this conflict, we conducted an intervention study where 26 participants underwent a callus abrasion while an age‐matched control group (*n* = 16) received no treatment. Skin hardness and thickness as well as vibration perception thresholds and touch sensitivity thresholds were collected before and after the intervention. The Callus abrasion significantly decreased skin properties. The intervention group exhibited no change in vibration sensitivity but had significantly better touch sensitivity. We argue that touch sensitivity was impeded by calluses because hard skin disperses the monofilament's standardized pressure used to stimulate the mechanoreceptors over a larger area, decreasing indentation depth and therefore stimulus intensity. However, vibration sensitivity was unaffected because the vibrating probe was adjusted to reach specific indentation depths, and thus stimulus intensity was not affected by skin properties. Since objects underfoot necessarily indent plantar skin during weight‐bearing, calluses should not affect mechanosensation during standing, walking, or running.

## Graphical abstract

This study researches the effect of a skin abrasion at the foot sole on cutaneous sensitivity. Results show that light touch sensitivity was diminished by hard and thick skin but vibration sensitivity was unaffected.

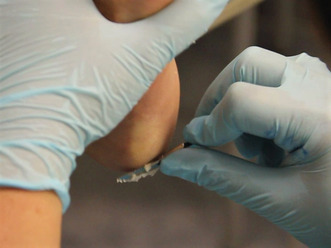

## 1 INTRODUCTION

Humans have been walking bipedally for at least 6–7 million years (Galik et al., 2004; Zollikofer et al., 2005). During bipedalism, the feet form the only physical contact with the ground, making them an important sensory organ for balance tasks and postural control (Viseux, 2020). Appropriately, the soles of human feet are densely packed with cutaneous mechanoreceptors (5–23 receptors/cm^2^), which transduce mechanical stimuli via action potentials to the central nervous system (Roll et al., 2002).

Generally, mechanoreceptors throughout our skin are the main information source for the perception of touch. However, afferent signals from foot sole receptors additionally contribute to upright body posture (Kavounoudias et al., 2001; Roll et al., 2002) and influence our perception of verticality (Foisy & Kapoula, 2018). They regulate postural sway (Nurse & Nigg, 2001) and trigger cutaneous reflexes in lower limb muscles, which adjust for balance disturbances (Aniss et al., n.d.; Fallon et al., 2005; Germano et al., 2016). Through natural aging processes, receptors deteriorate structurally and decline in number, resulting in decreased cutaneous sensitivity (Shaffer & Harrison, 2007). Similar effects are caused by neurological disorders (Paré et al., 2007; Prätorius et al., 2003), which ultimately lead to an increased risk of falls, especially in elderly people (Bretan, 2012; Kerr et al., 2010; Meyer et al., 2004). Decreasing mechanoreceptor sensitivity artificially, by cooling or anesthesia of the foot sole, has also been shown to change gait patterns (Eils et al., 2002; McDonnell & Warden‐Flood, 2000; Taylor et al., 2004) and muscle activity during gait (Eils et al., 2004; Nurse & Nigg, 2001), impairing postural control. Concurrently, enhancing tactile feedback through textured surfaces or vibrating insoles has led to improved response ability to unexpected perturbation during gait termination and reduced sway and gait variability (Chen et al., 2020; Lipsitz et al., 2015; Robb et al., 2021).

**FIGURE 1:**
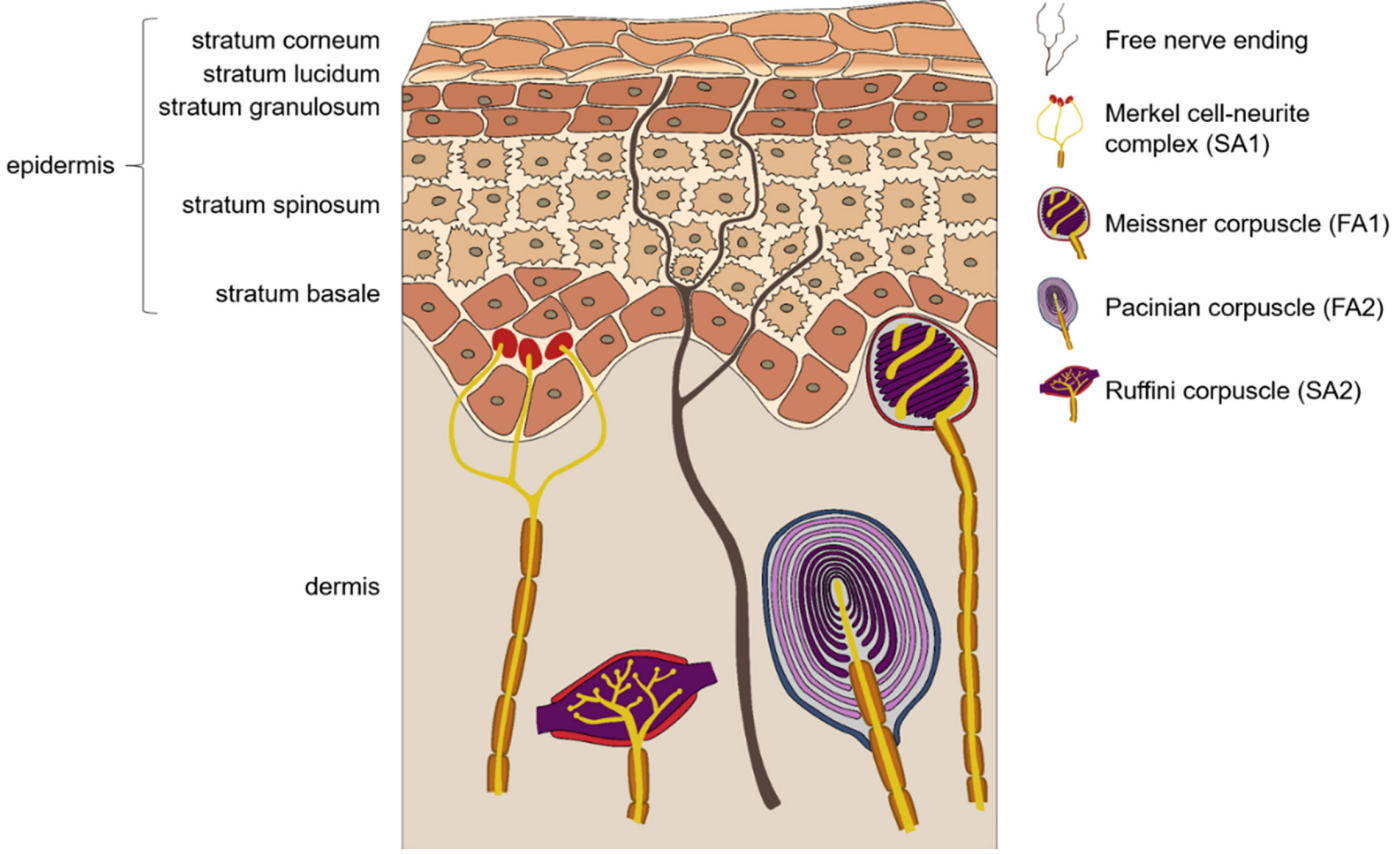
Cutaneous Mechanoreceptors in plantar skin. While free nerve endings and Merkel cell‐neurite complexes reach into the epidermis, the encapsulated, fast adapting Meissner, and pacinian corpuscles lie in the dermis.

Plantar cutaneous information originates from four classes of mechanoreceptors, which can be distinguished by their receptive fields (types 1 and 2) and ability to adapt quickly (FA) or slowly (SA) to continuous indentation. SA1 and FA1 have small, clear‐cut receptive fields and can be found in the basal epidermis and the superficial dermis, while SA2 and FA2 have large, obscure fields and lie deeper in the dermis (Johansson, 1978; Johnson, 2001; Figure 1). SA receptors sense prolonged pressure and stretch (Johansson & Vallbo, 1983; Macefield et al., 1990), providing information about persistent stimuli, like the pressure distribution between the feet and ground during stance. FA receptors account for the majority (60%) of foot sole mechanoreceptors (Strzalkowski et al., 2018) and act like differential sensors, coding for dynamic events and sudden changes (Perry et al., 2000). Two methods are frequently used in science and healthcare to evaluate receptor performance in a noninvasive way (Gandhi et al., 2011; Tan, 2010). Vibration perception thresholds (VPT), raised at the frequency range of FA1 (5–50 Hz) or FA2 receptors (100–300 Hz), evaluate the respective receptor sensitivity by recording the smallest perceived vibration (Johansson & Vallbo, 1983; Löfvenberg & Johansson, 1984). Similarly, monofilament thresholds (MT) determine the smallest perceived pressure stimulus by gently applying nylon filaments of varying sizes to the skin. Although MTs and VPTs impose very different stimuli on the skin, both methods are detected predominantly by FA receptors and therefore are used to evaluate these receptors' sensitivity (Strzalkowski, Mildren, & Bent, 2015). However, all stimuli must travel through the intermediate skin before reaching the receptor. Therefore, mechanical characteristics of the skin inevitably play a role in stimulus transmission—a topic that is discussed in recent research (Holowka et al., 2019; Strzalkowski, Triano, et al., 2015; Zippenfennig, Wynands, et al. 2021). Interestingly, these skin properties are not static but can adapt when confronted with mechanical stress (Sanders et al., 1995).

High friction triggers the formation of a protective thickening of the outer layer of the epidermis, which is sometimes referred to as “callus” (Sanders et al., 1995). This process involves the hyperproliferation of keratinocytes in the deepest layer of the epidermis. As these cells migrate outward, they maintain a more voluminous, nonsquamous shape than usual. Conjointly with increased expression of adhesion molecules and decreased hydration, a callus is formed that is both thicker and harder than normal skin (Hashmi et al., 2015; Kim et al., 2010). Theoretically, thicker skin widens the gap between stimulus and mechanoreceptor, while increased hardness could possibly disperse stimuli over a larger area. Both changes may alter incoming stimuli during transmission. To date, only a few studies have investigated the influence of skin properties on foot sole sensitivity, yielding conflicting results. Jammes et al. (2017) compared vibration sensitivity at three plantar foot regions and found the lowest sensitivity at the location with the greatest hardness (5th Met). Additionally, in a small subset of subjects (*n* = 6), they observed greater vibration sensitivity when skin hardness was reduced through skin abrasion (Jammes et al., 2017). In contrast to these results, other studies found no significant influence of skin properties on vibration sensitivity across subjects or between foot regions (Holowka et al., 2019; Strzalkowski, Triano, et al., 2015). However, to confuse things further, Strzalkowski, Mildren, et al. (2015) and Strzalkowski, Triano, et al. (2015) reported a small but significant effect of skin hardness on sensitivity measured using monofilaments, suggesting that skin properties may affect only specific stimuli. These conflicting results indicate the need for further research on the dynamics between skin properties and sensitivity thresholds. Accordingly, this study investigates whether the abrasion of calluses alters skin properties and influences both monofilament and vibration sensitivity at 30 and 200 Hz in a large subject sample. Based on previous studies, we predict that the abrasion will decrease hardness and thickness and cause an improvement in monofilament sensitivity while vibration sensitivity remains unchanged (Holowka et al., 2019; Strzalkowski, Triano, et al., 2015). This study addresses conflicting past research on skin properties and sensitivity and tests whether callus abrasion, which is often implemented in treating diabetic foot syndrome to lower pressure hotspots (Pataky et al., 2002), also benefits sensitivity.

## 2 MATERIALS AND METHODS

### 2.1 Participants and ethical approval

Forty‐two healthy participants (21 ♂/ 21 ♀; mean ± SD: 49.7 ± 11.5 years, 76.1 ± 15.4 kg, 170.8 ± 11.2 cm) were acquired through multiple calls for subjects in the local press, provided written informed consent, and took part in the study. To be included, participants had to self‐report the presence of plantar calluses and the absence of any diseases which could affect sensory performance, like diabetes.

### 2.2 Experimental design

**FIGURE 2:**
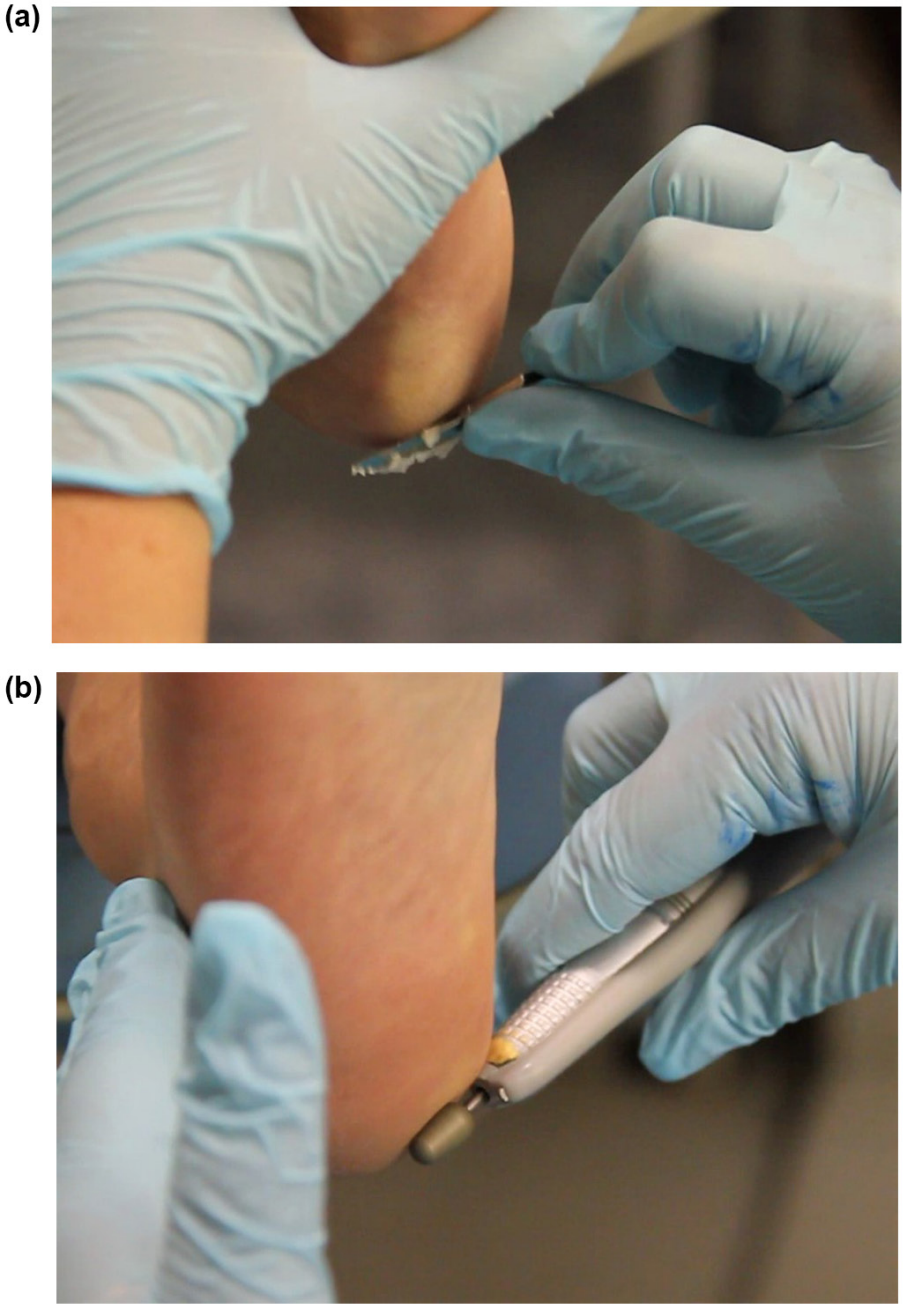
Skin abrasion procedure: (a) After the application of a skin softening lotion, a scalpel was used to carefully remove callus tissue layer by layer. (b) A burr was used to smooth out the remaining irregularities.

To investigate the effect of calluses on mechanosensitivity, we examined mechanical and sensory aspects of the foot sole before and after a professional pedicure, in which a professional podiatrist abraded callused skin using a scalpel and burr (Figure 2a,b). The study features an intervention group (IG; *n* = 26) and a control group (CG; *n* = 16). The control group was acquired in a second recruitment phase, underwent no treatment, and was matched for age. The most calloused plantar spot, detected by visual examination and palpation, served as the measurement site. Accordingly, 38 participants were measured in the heel region and 4 at the metatarsal heads. The pre‐ and posttests were identical and consisted of sensory and mechanical examinations. All measurements were performed by a single investigator under laboratory conditions. To prevent acute side effects like skin irritation from influencing sensory examination, in the intervention group the postmeasurement was conducted at least 24 h after the skin abrasion.

### 2.3 Sensory examination of the foot sole

Initially, participants underwent a 10‐min acclimatization period with bare feet to reduce temperature fluctuation during data collection. Measurements were conducted in the prone position. The foot was supported perpendicularly to the slightly propped‐up lower leg. VPTs, defined as the smallest perceivable vibration amplitude, were taken using a customized vibration exciter (Mini‐Shaker, Brüel & Kjaer Vibro; type 4180). To emit target amplitudes reliably, the device was initially calibrated by a high‐precision capacitive displacement sensor (CS05, Micro‐Epsilon Messtechnik GmbH & Co. KG). During testing, the output amplitude of a probe with a rounded tip (7.8 mm diameter) was documented using an acceleration sensor (MMA2240KEG, Freescale Semiconductor). An integrated force sensor (DS050A9, Disynet GmbH) monitored the force between probe and skin, which was maintained between 0.7–1.2 N (Holowka et al., 2019; Zippenfennig, Wynands, et al., 2021). Participants wore headphones (QuietComfort 25, Bose) to eliminate environmental noise. We chose 30 and 200 Hz to measure FA1 and FA2 receptors. These frequencies lie in the middle of their respective sensor ranges and maximize the chance of exclusively stimulating the target receptor (Johansson & Vallbo, 1983; Löfvenberg & Johansson, 1984). Following a customized threshold protocol, participants were instructed to press a handheld button whenever they perceived a 2‐s vibration burst (Holowka et al., 2019). Between bursts, randomized breaks (2–7 s) ensured that an upcoming burst could not be anticipated based on time. After an initial vibration was perceived, the amplitude was continually halved until participants were unable to detect a burst. The amplitude of subsequent vibration was the midpoint between the smallest perceived and the largest unperceived burst. Each subsequent burst became a limit for the following one, systematically narrowing the threshold location. The protocol ended four bursts after the first unperceived vibration. The VPT was calculated as the mean of the smallest perceived and the largest unperceived vibration amplitude in micrometers. Three trials per frequency were recorded in randomized order and their means were used for data analysis.

Light touch sensitivity was measured using a full set of 20 tactile monofilaments (Fabrication Enterprises Inc.) calibrated to 0.008–300 g target force. Filaments were applied slowly and perpendicularly to the skin until they bent into a c‐shape. After 1 s, the monofilament was lifted from the skin. Participants were instructed to verbally signal each time they perceived a pressure stimulus. We used a customized 4–2‐1 stepping algorithm to acquire MT (Snyder et al., 2016). The initial measurement started with the 10 g filament. One measurement always consisted of 5 filament‐skin contacts, with 2–3 of these being null stimuli to register false positive answers. After at least four out of five applications were detected correctly, the filament for the next measurement was decreased by four sizes. Otherwise, the filament was increased by four sizes. Whenever an unperceived measurement followed a perceived one or vice versa, a reversal point was reached. After the first reversal, the size change between measurements was reduced from 4 to 2. After the second, sizes only changed by 1 and the third reversal point ended the protocol. The last perceived measurement was recorded as MT and used for data analysis.

### 2.4 Mechanical properties of the foot sole

Skin hardness, determined according to indentation depth of steady pressure, was measured using a Shore OO Durometer (Checkline, Cedarhurst). Participants lay prone with the knee supported in 90° flexion and the foot perpendicular to the lower leg. This position was monitored carefully because any alteration could affect durometer readings by changing the skin tension. The durometer probe (diameter, 2.4 mm) was applied perpendicularly and only with the device's own weight (exerted force: 1.111 N). Based on the indentation depth of the probe, the analog scale presented values between 0 (softest) and 100 (hardest) Shore units (Sh). A reading was taken 10 s after the probe was applied and there was a 20 s pause before the next measurement to prevent the accumulation of skin indentation. Three readings were recorded, and the mean was used for data analysis.

Epidermal thickness was measured using an ultrasound scanner (GE LogiQ A5, Ge‐nereal Electric Company) connected to a linear array probe (GE 11 L, General Electric Company), which produced videos in B‐mode at 12 Mhz and 2 cm image depth. Epidermal thickness was measured last to prevent skin softening through ultrasound gel from affecting durometer readings (Schmidt et al., 2018). Participants lay prone with the lower legs supported in a slightly elevated position, and the probe was placed perpendicularly and with minimal force to avoid skin compression. All ultrasound recordings were assessed frame by frame. The image with the most visible borders of the epidermis was used for further analysis. Epidermal thickness was measured using ImageJ (National Institutes of Health, Washington D.C., Maryland, USA) and defined as the distance between the two hyperechoic layers that demarcate the borders of the epidermis (Wortsman, 2021). The reading was taken at the area in the image that depicted the hyperechoic layers most distinctly. To avoid measurement bias, all images were assigned random identifier numbers and measured without the investigator knowing which individual or block (pre and post) the image originated from.

### 2.5 Statistical analysis

Outliers larger than ±3 SD from the mean (three VPT in IG and one MT in CG) and ultrasound images with low quality (three in IG) were eliminated from the data set (Strzalkowski, Triano, et al., 2015). Sensitivity and skin parameters were checked for the benefit of an Ln transformation (Schmidt et al., 2019). All parameters, except MTs, exhibited normality and homoscedasticity after these transformations (Schmidt et al., 2019). *T* tests and Mann–Whitney U tests were used to check for initial group and gender differences. Significant pretest group differences were found for skin hardness and touch sensitivity, which influenced the choice of our subsequent statistical approach. There were no differences found in the gender data, so they were pooled for the statistical analyses (SPSS 28, IBM).

Initially, we conducted regression models for each skin property and VPT frequency. Posttest values were used as dependent variables. The first model only used the pretest value as a covariate. The second model used only the group factor, while the third model used both pretest values and group factors. The fourth model was similar to the third but included an interaction term. To test the different factors, ANOVA was used for model comparisons (Table S2). Subsequently, we performed an analysis using mixed ANOVAs of change. This method only assumes the equality of change, which potentially introduces less bias in studies with preexisting group differences (van Breukelen, 2006). Subsequently, the intervention effect on skin properties and vibration sensitivity was assessed using mixed ANOVAs of change (Table S1). Group was the between‐subject factor and time (pretest vs. posttest) was the within‐subject factor. Equality of covariances was checked using box tests and main effects were analyzed for both factors and their interaction. Unfortunately, covariance proved to be unequal in skin thickness and hardness. As an alternative solution, one‐way ANOVAs of change were conducted for each group separately to analyze pre–post differences and their respective effect sizes (Table S1).

Finally, we used general linear models (GLM) to assess the relationship between vibration sensitivity and the effects of skin abrasion. Pretest values, skin hardness, and skin thickness differences were set as covariates and group (IG or CG) as fixed effects (Table S3). Due to nonnormality and the nonmetric scale of MTs, we used descriptive analysis and a Wilcoxon signed‐rank test to check for intervention effects. Spearman rank correlations were used to investigate the relationship between skin properties and MTs.

## 3 RESULTS

The plantar temperature was consistent throughout all measurements (VPT pre vs. post: 27.5 ± 2.7°C vs 28.1 ± 2.5°C; MT pre vs post: 27.5 ± 2.8°C vs 28.0 ± 2.5°C). IG pretests were significantly higher than CG pretests for skin hardness (*p* < 0.001, *r* = 0.508) and MTs (*p* < 0.001; *r* = 0.616). Similar trends were found for skin thickness and 30 Hz VPTs but were not significant (Table 1).

**TABLE 1:** Skin properties and sensitivity measures before and after the intervention (MW ± SD)

	Thickness (mm)	Hardness (Sh)	30 Hz VPT (μm)	200 Hz VPT (μm)	MT (g)
IG					
Pre	1.00 ± 0.29	43.02 ± 10.23	21.87 ± 15.67	1.84 ± 1.60	31.40 ± 26.63
Post	0.84 ± 0.23	37.60 ± 6.73	19.87 ± 13.33	1.90 ± 1.83	13.85 ± 15.40
CG					
Pre	0.92 ± 0.16	32.38 ± 7.84	15.00 ± 11.23	1.73 ± 1.50	7.48 ± 14.82
Post	0.92 ± 0.18	32.75 ± 8.05	14.92 ± 9.98	2.01 ± 1.74	10.77 ± 24.90

**FIGURE 3:**
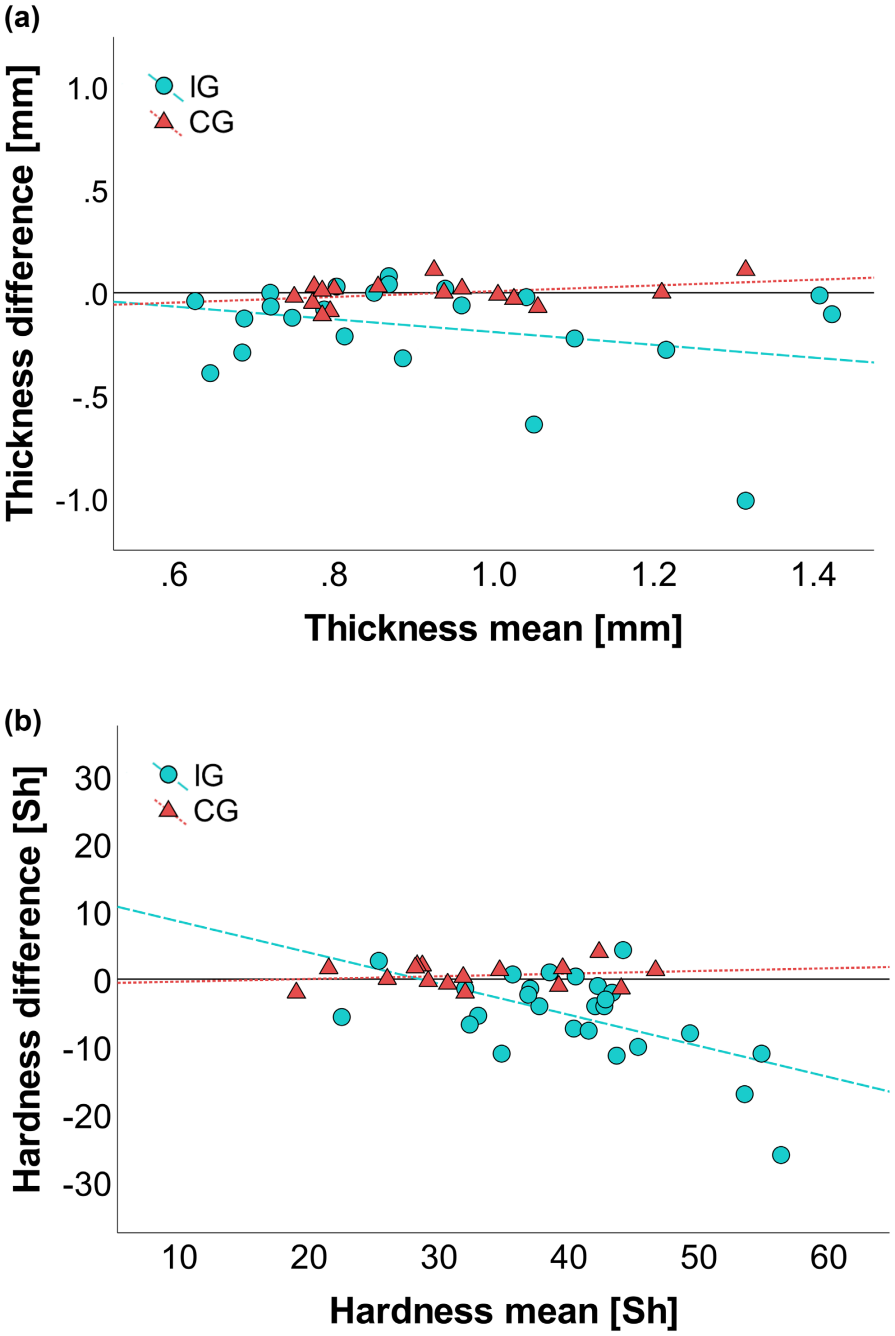
Intervention effects on skin properties: (a) Difference plot of skin thickness (*n* = 23 IG, 16 CG). (b) Difference plot of skin hardness (*n* = 26 IG, 16 CG). Dashed lines represent an indicator for the best linear fit for each group. The continuous lines mark the zero value.

The intervention reduced IG thickness significantly (*p* = 0.002, *η* ^2^ = 0.367) by 0.17 ± 0.25 mm (17%), while CG did not exhibit any significant changes between time points (Table 1). The treatment effect was not uniform across the IG, since thickness only decreased in 65.4% of the participants, with changes ranging from 0.08 to −1.01 mm. The difference plot reveals a slight tendency for individuals with initially thick skin to produce larger changes (Figure 3a). This trend was more consistent for skin hardness, where initially hard skin, especially at values above 40 Sh, consistently led to large treatment changes (Figure 3b). Overall, skin hardness in IG was significantly (*p* < 0.001, *η* ^2^ = 0.468) reduced by 5.42 ± 6.69 Sh (12.5%), while CG values remained constant between time points (Table 1). The treatment effect was more uniform for hardness, as 80.8% of IG showed hardness reductions, with individual changes ranging from 4.3 to −26 Sh (Figure 3b). Furthermore, initial hardness correlated significantly (*p* = 0.005, *r* ^2^ = 0.194) with initial thickness across both groups.

**FIGURE 4:**
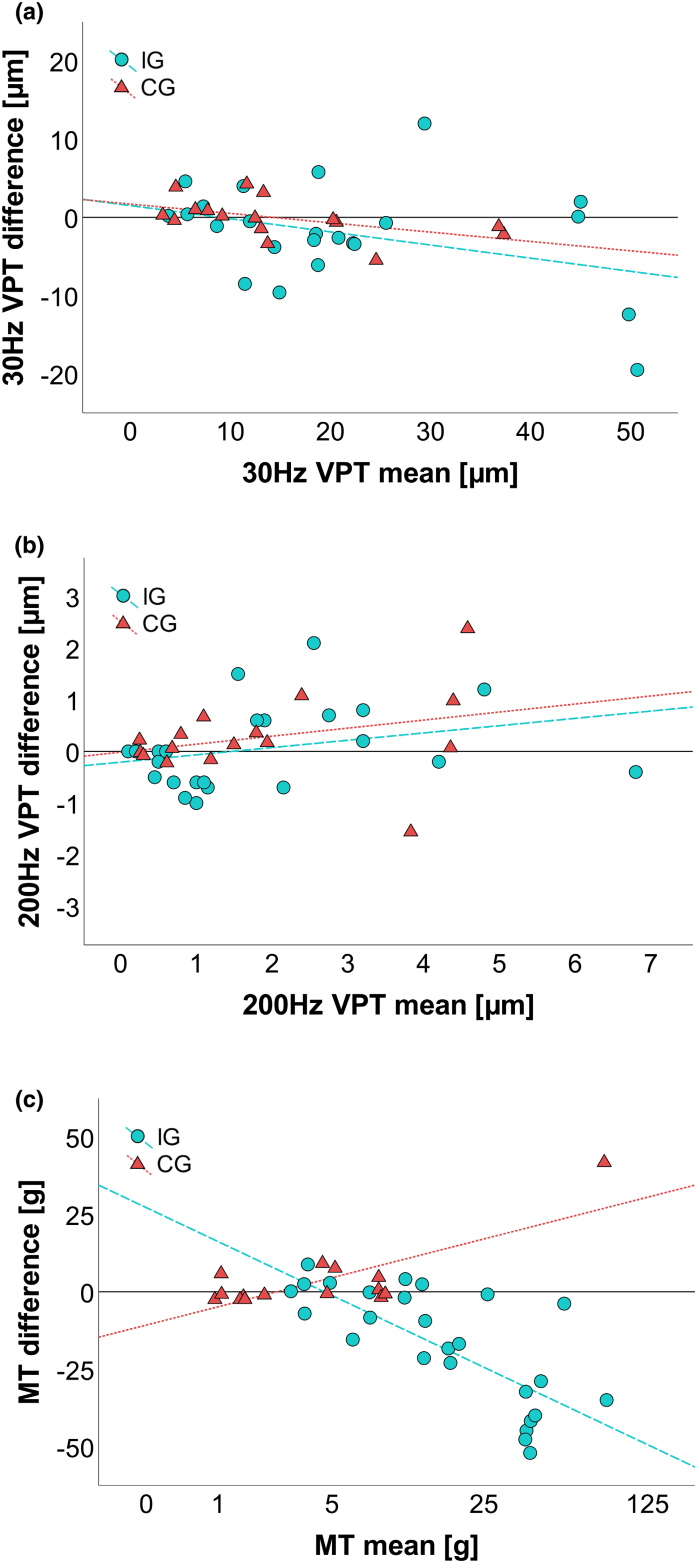
Intervention effects on sensitivity parameters: (a) Difference plot of 30 Hz VPTs (*n* = 23 IG, 16 CG). (b) Difference plot of 200 Hz VPTs (*n* = 23 IG, 16 CG). (c) Jittered difference plot of MTs (*n* = 26 IG, 15 CG) to uncover overlapping data points. Dashed lines represent an indicator for the best linear fit for each group. The continuous lines mark the zero value.

**FIGURE 5:**
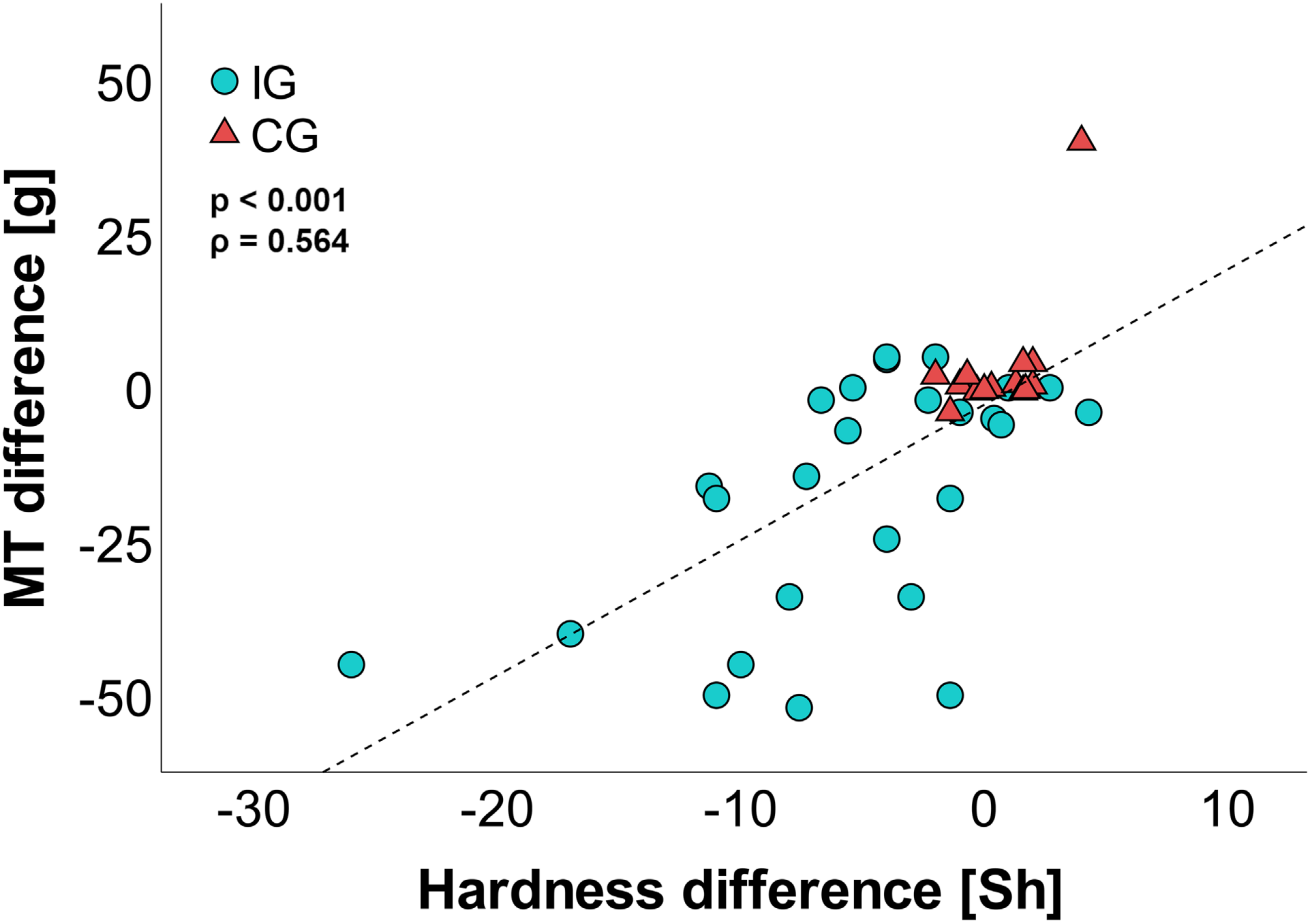
Relationship between monofilament thresholds (MT) and skin hardness. Scatter plot of MT differences vs skin hardness differences (*n* = 23 IG, 15 CG). Dashed lines represent an indicator for the best linear fit over all participants. A significant Spearman correlation (*p* < 0.001, *ρ* = 0.564) was found using untransformed data.

After the intervention, no significant changes were found for either 30 or 200 Hz in both groups (Figure 4a,b), This result was verified by the comparisons of various regression models (Table S2) as well as ANOVAs (Table S1), which yielded no significant main effects for group or time. GLMs showed that neither hardness nor thickness differences had a significant effect on vibration thresholds at 30 or 200 Hz (Table S3). A slight decrease of 2.00 ± 6.48 μm (9.1%) at 30 Hz in the IG was mainly caused by two participants with high initial threshold values (Figure 4a). MTs decreased significantly by 17.55 ± 19.59 g (55.9%) (*p* < 0.001, *r* = 0.687). This drop was consistent in the majority of IG subjects (76.9%; Figure 4c). CG exhibited an increase of 3.29 ± 10.35 g, which failed to be significant, as it was largely produced by a singular data point (Figure 4c). Across both groups, pre–post differences of MT correlated with hardness differences (*p* < 0.001, ρ = 0.564), but not with thickness differences (Figure 5).

## 4 DISCUSSION

The aim of this study was to investigate the influence of skin mechanics on vibration and touch sensitivity through callus abrasion. We hypothesized that decreased thickness and hardness would result in no change in vibration sensitivity but would boost touch sensitivity.

Evaluating vibration sensitivity, no initial differences were found between groups. As hypothesized, the intervention did not change either 30 or 200 Hz thresholds. These findings are in line with the results of Strzalkowski, Mildren, et al. (2015) and Strzalkowski, Triano, et al. (2015) and Holowka et al., 2019 and strengthen the hypothesis that normal plantar calluses do not influence vibration perception. The fact that Jammes et al. (2017) found conflicting results through skin abrasion might be linked to the exceptionally high initial skin hardness (70 Sh) of their sample, which consisted of six participants with higher hardness values than any of the subjects in our study. This sample generated a remarkable decrease of 26 Sh after the intervention and demonstrated that extreme skin hardness can impede vibration sensitivity. However, previous studies show that average plantar skin hardness in shod populations lies between 28 and 46 Sh over various plantar foot regions (Table S4). Even habitually barefoot cohorts only had averages of 40–54 Sh (Table S4). Therefore, the Jammes et al. sample reflects neither the physiological norm nor the usually achievable effect following skin abrasion.

One possible explanation for the absence of any changes in VPTs could be that diverging factors canceled each other out. In theory, thicker skin increases the travel distance to the receptor, decreasing sensitivity (Jajor et al., 2019). On the other hand, increased skin hardness may decrease damping capabilities, so that vibrations travel further. In addition, hard and stiff skin might transmit 200 Hz stimuli better, since its resonant frequency is thought to be higher and therefore closer to 200 Hz than the resonant frequency of normal skin, which lies at 140–185 Hz (Panchal et al., 2019). Based on these considerations, high thickness could decrease sensitivity and high hardness could increase sensitivity. However, neither property had a significant effect on sensitivity in the GLM, which suggests that they did not cancel each other out but were negligible. Alternatively, the absence of VPT changes might be caused by the characteristics of the stimulus. VPTs are standardized to the vibration amplitude, which reflects the indentation depth of the probe. Therefore, despite high thickness or hardness, VPTs cannot be repelled and will always indent the skin. Since VPT amplitudes measure only a few microns, it seems that even callused skin, once indented, shows little to no damping capabilities before receptors are reached. Therefore, the receptors might receive all stimuli that manage to indent the skin without the loss of information. Like VPTs, almost all objects underneath our feet impose an indentation in real‐world circumstances. However, because these objects are trapped between the foot and the ground, their pressure and the resulting indentation in most cases exceed those of the VPT probe. Since skin properties did not affect the relatively small VPT amplitudes, it is unlikely that they will affect larger stimuli. Therefore, calluses should not have any significant effect on sensing the ground and therefore no negative effect on postural control during dynamic behaviors.

Monofilament thresholds, examined via Wilcoxon signed‐rank tests, showed significant changes of 17.55 ± 19.59 g (55.9%; *p* < 0.001, *r* = 0.687) between pre‐ and posttests in IG, while no changes were present in CG. We therefore conclude, in agreement with recent literature, that monofilament thresholds are affected by skin properties (Strzalkowski, Mildren, et al., 2015; Strzalkowski, Triano, et al., 2015). This result is interesting since both VPTs and MTs are conveyed by the same FA receptors but do not produce the same outcome (Strzalkowski, Mildren, et al., 2015). The obvious difference between both methods is that one is a high‐frequency vibration, whereas the other is a single touch with a filament that is applied for 2 s and then withdrawn. However, continuous vibration in VPTs and static duration in MTs might not play a crucial role in threshold detection, as testing in both methods showed that onset and offset are the key moments at which these stimuli are detected. This finding is in line with general excitability patterns of FA receptors and is the reason why we focused on each method's onset and offset moments (Johansson & Vallbo, 1983). The crucial difference between the stimuli is that VPTs were standardized to the indentation depth, while monofilaments were standardized to the pressure they apply. If the force of a filament is small, it may only indent the skin marginally, resulting in a small stimulus at the receptor level. High hardness might impede an indentation as well, effectively damping a stimulus. This idea is strengthened by the observation that harder skin formed flatter indentation craters than soft skin (Figure S1) as well as the correlation between skin hardness and MT values (Figure 5). In essence, calluses act like a shell that disperses a filament's point pressure over a larger surrounding area. While potentially more receptors can be reached through this process, all receptors would be struck with a lower force. Because the forces in question already operate at the perceptive threshold levels, the dispersion potentially decreases them to a subthreshold level, effectively increasing monofilament thresholds in hard skin. Although skin thickness could theoretically impede the transmission of monofilament stimuli by increasing the distance between stimulus and receptor, the absence of a correlation between the two parameters conveys that this effect was negligible in our study. However, other literature found a significant relationship between thickness and MTs (Strzalkowski, Triano, et al., 2015). Therefore, more research is necessary on the separate effects of both parameters.

While our results support our predictions for the effects of the skin abrasion on both MTs and VPTs, we were surprised to observe inconsistent effects of this intervention on skin properties. IG skin thickness significantly decreased by 0.17 ± 0.25 mm (17%) and hardness significantly decreased by 5.4 ± 6.5 Sh (12.5%), while CG showed no significant differences. However, these treatment effects exhibited large variations. Some participants produced changes up to −1.01 mm in thickness and −26 Sh in hardness. Others showed little to no change but were kept in the sample to give a realistic measure of the effect of skin abrasion. One reason for the large variation is the nature of the treatment. It is evident that skin abrasion is most effective when the recipient has a large amount of callus and has less effect, on diminutive calluses. This proportional relationship is visible in the hardness values, where large initial values led to large changes (Figure 3b). Since the sample included participants with hard and soft skin, the treatment effect varies substantially. A second reason for the variation specifically concerns skin thickness. Here, the relationship between initial value and treatment effect is less clear than for skin hardness (Figure 3a). Unexpectedly, a few participants with high thickness values did not exhibit large changes in thickness after the intervention, increasing the variation further. In those subjects, we observed that thick skin did not occur in the form of large calluses, but rather as normal skin with little abradable tissue. These, and other small calluses below 0.1 mm in thickness may have been too small to detect by the ultrasound device. Prior research has shown that epidermal thickness varies in glabrous skin interindividual and with age (Lavker et al., 1987, 1989). The same might be true for plantar skin and could explain some of the variations in skin properties of these participants, although we did not find a significant effect of age on skin thickness in our sample. On a side note, these results show that hardness seems to be a better indicator of callus formation than epidermal thickness. An additional possible cause of variation in the intervention effect could have been the location of the measurements. While most subjects were measured at the heel, a few were measured at the metatarsal region, because this was the most callused region on the foot. The abrasion also had to be performed more conservatively in subjects whose foot soles had large cracks due to high skin brittleness, providing another possible source of intervention variation.

Even though these factors resulted in a large variation in intervention effectiveness, the one‐way ANOVAs of change results verified significant changes in hardness and thickness, and therefore verified the effectiveness of the intervention. In recent literature, only Jammes et al. (2017) abraded the skin in a small sample (*n* = 6) with extremely high initial skin hardness (69 Sh). His intervention lowered hardness by ca. 25 Sh, proving the assumption that abrasion leads to more pronounced changes in people with very hard skin. Other studies compared skin properties between different cohorts or foot regions and exhibited differences up to 0.6 mm in thickness and 11.7 Sh in hardness (Holowka et al., 2019; Strzalkowski, Triano, et al., 2015). Our study design did not achieve within‐subject changes of that magnitude. However, because of its intervention design, the results were not affected by variance possibly arising from receptor density differences between foot regions or interindividual differences. Since pre and postdata were collected at the same location, regional differences in receptor density (Strzalkowski et al., 2018), which are expected to influence vibration sensitivity particularly at frequencies under 40–60 Hz (Löfvenberg & Johansson, 1984), did not affect our results. Furthermore, interindividual sensitivity differences, which increase at high vibration frequencies (Löfvenberg & Johansson, 1984), did not influence the pre–post design. Therefore, any detected sensitivity changes were related to the treatment.

The initial skin properties of the sample were in line with prior research, which reported a range of 0.5–1.4 mm in thickness and 25–60 Sh in hardness for healthy, habitually shod populations (Chao et al., 2011; Holowka et al., 2019; Jammes et al., 2017; Strzalkowski, Triano, et al., 2015; Zippenfennig, Drechsel, et al., 2021). Because this study specifically recruited participants with plantar calluses, our values are presumably greater than the mean of shod populations of the same age in Germany and other industrialized countries. Initial MTs, particularly in IG, was higher and therefore indicate less sensitivity than the values of 2.5–6.6 g documented so far in the heel region (Billot et al., 2013; Jain et al., 2008; Perry, 2006; Song et al., 2021). Since the existence of callused skin is the main difference between our sample and prior study samples, a relationship between monofilament sensitivity and skin properties is a potential explanation for this offset. VPTs reiterated the value range of studies researching normal feet, suggesting that preexisting calluses in our sample did not diminish vibration sensitivity (Chao et al., 2011; Holowka et al., 2019; Zippenfennig et al., 2018). When comparing groups, significantly higher values in skin hardness and MTs were measured for IG, hinting at a relationship between those parameters. Similar trends were found for skin thickness. The reason for these preexisting group differences can only be chance since both groups were recruited in the same way.

## 5 CONCLUSION

In summary, the abrasion of calluses effectively decreased skin properties and affected MTs, but not VPTs. The fact that VPTs were unchanged suggests that skin properties in a physiological range do not dampen stimuli after they indent the skin. Because objects under the feet indent the foot sole with the force of our body weight and by larger margins than produced during vibration tests, we think that most plantar stimuli during walking and standing will not be impaired by skin properties. As a result, we expect that dynamic balance should not be affected by skin properties in normal ranges, including among people who are regularly barefoot (Holowka et al., 2019). However, based on differences between our findings and those of Jammes et al. (2017), we suggest that vibration sensitivity might be enhanced by skin abrasion in cases of exceptionally thick callus formations. In such cases, abrasive treatments can achieve large reductions of skin thickness and hardness, which may improve pressure distribution under the foot and increase sensitivity in a manner that could improve balance.

MTs correlated significantly with skin hardness, which indicates that hard skin flattens or repels the indentation of very light stimuli, resulting in decreased touch sensitivity. While decreased sensitivity to touch might be detrimental to task performance in our hands, there are only few scenarios where small, fleeting pressure stimuli like monofilaments stimulate our feet. Due to the load of our bodyweight, even a small stone inside a shoe causes pressure that indents the skin and is therefore more comparable to a VPT stimulus than an MT stimulus. For these reasons, VPTs bear more meaningful information for the functional sensitivity of the foot sole than MTs. Since VPTs are not affected by skin properties, they are likely to be the best choice to analyze sensory deterioration in diabetic feet, where skin property changes could skew data of devices like monofilaments.

In contrast to other studies, hardness and thickness showed only a weak correlation in our sample (Holowka et al., 2019; Strzalkowski, Triano, et al., 2015). This unexpected result forced us to consider their separate effects, concluding that skin hardness might be the crucial factor in decreasing monofilament sensitivity. Further research should focus on separate effects of hardness and thickness on various stimuli and their transmission through shoe soles as well as other sensory information such as pain. Future scientific results could contribute to optimizing minimal shoes in terms of comfort and sensitivity preservation.

## AUTHOR CONTRIBUTIONS

T.L.M conceptualized and supervised the research process. C.Z. contributed to the statistical analysis and visualization. B.W. lead the investigation and wrote the original Draft. N.B.H. contributed to the writing of the discussion and conclusion and guided the review and submission process. All authors contributed to the review and editing of the article.

## Funding information

This research received no specific grant from any funding agency in the public, commercial, or not‐for‐profit sectors.

## CONFLICT OF INTEREST

The authors declare that they have no conflict of interest.

## INSTITUTIONAL REVIEW BOARD STATEMENT

The study was conducted in accordance with the Declaration of Helsinki and approved by the Ethics Committee of Chemnitz University of Technology (V‐294‐17‐TM‐Fußsensibilität‐12102018).

## INFORMED CONSENT STATEMENT

Informed consent was obtained from all subjects involved in the study.

## Supporting information


Figure S1a

Figure S1b

Table S1:
