# Supplementary figures and images for: Does plantar skin abrasion affect cutaneous mechanosensation?
Source: Physiol Rep. 2022 Oct 18;10(20):e15479. doi: 10.14814/phy2.15479 (PMC9579735; doi:10.14814/phy2.15479)

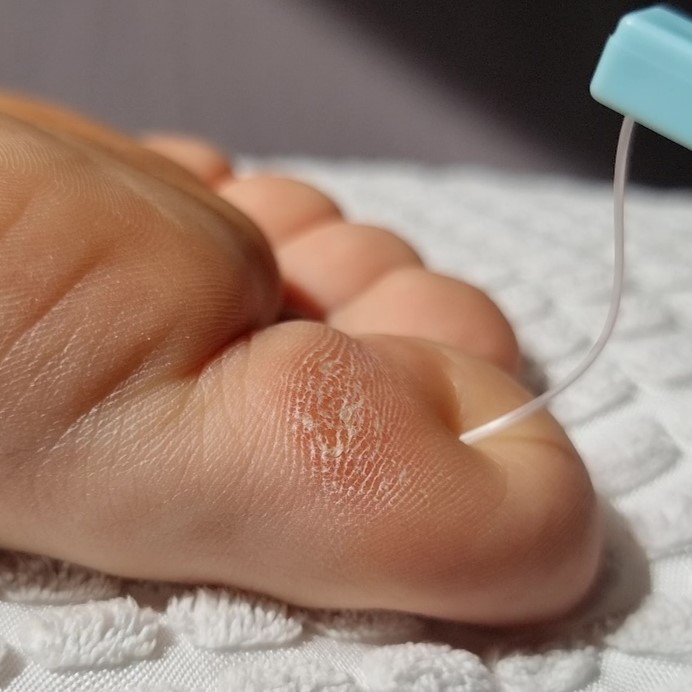

Supplement: Supplementary file 1 — Figure S1a [file PHY2-10-e15479-s003.jpg]

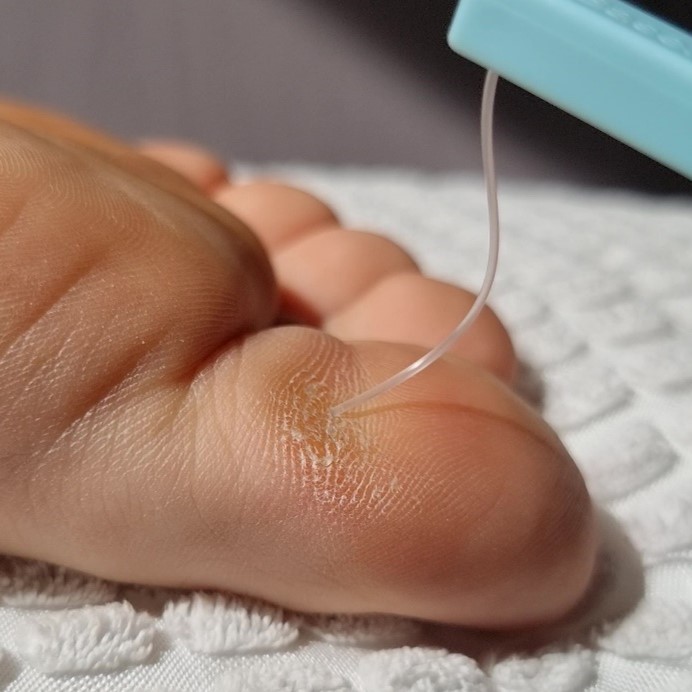

Supplement: Supplementary file 2 — Figure S1b [file PHY2-10-e15479-s002.jpg]
