# Supplementary material for: Does plantar skin abrasion affect cutaneous mechanosensation?
Source: Physiol Rep. 2022 Oct 18;10(20):e15479. doi: 10.14814/phy2.15479 (PMC9579735; doi:10.14814/phy2.15479)
Supplement: Supplementary file 3 — Table S1: [file PHY2-10-e15479-s001.docx]

**Supplement Tables**

**Table S1:** Mixed ANOVAs of vibration sensitivity and one way ANOVAs of skin properties.

| **Parameter** | **Statistic** | **Factor** | **F-distribution** | **F-statistic** | **P-value** | **η²** |
| --- | --- | --- | --- | --- | --- | --- |
| 30Hz VT | Mixed ANOVA | Group | 1;37 | 2.072 | 0.158 | 0.053 |
|  |  | Time | 1;37 | 0.014 | 0.905 | 0.000 |
|  |  | Interaction | 1;37 | 1.373 | 0.249 | 0.036 |
| 200Hz VT | Mixed ANOVA | Group | 1;37 | 0.025 | 0.876 | 0.001 |
|  |  | Time | 1;37 | 0.002 | 0.968 | 0.000 |
|  |  | Interaction | 1;37 | 2.797 | 0.103 | 0.070 |
| Hardness | One way ANOVA | IG Time | 1;25 | 21.989 | <0.001 | 0.468 |
|  | One way ANOVA | CG Time | 1;15 | 1.049 | 0.322 | 0.065 |
| Thickness | One way ANOVA | IG Time | 1;22 | 12.729 | 0.002 | 0.367 |
|  | One way ANOVA | CG Time | 1;15 | 0.235 | 0.635 | 0.015 |

**Table S2:** ANOVA with model comparisons. The response variable in all models are the post-values of the respective parameters. The pre-values were centered in all models to improve the multicollinearity assumption. For "Group", the reference value is "CG". P-values are based on two-tailed type 3 ANOVAs. For skin hardness, the most complex model (interactive) is significantly better than the simpler models based on the model comparisons. For skin thickness, model 3 (additive) is the best fitting model. Both vibration threshold data is best predicted by model 1 (ln(Pre-Values)). Unfortunately, for skin hardness and thickness, the model assumptions (homoscedasticity and normal distribution of the residuals) are violated. Nevertheless, this analysis confirms the results of the ANOVA of change.

| **Parameter** | **Model** | **Predictor** | **Coefficient** | **Standard Error** | **F-value** | **P-value** |  | **Adjusted R-squared** | **Model comparisons** |
| --- | --- | --- | --- | --- | --- | --- | --- | --- | --- |
| Skin Hardness | ln(Pre-Values) | (Intercept) | 3.55 | 0.02 | 43277.17 | <0.001 |  | 0.77 | ln(Pre-Values) vs. Additive p = 0.03 |
|  |  | ln(Duro_Pre) | 0.74 | 0.06 | 140.26 | <0.001 |  |  |  |
|  | Group | (Intercept) | 3.46 | 0.06 | 3845.09 | <0.001 |  | 0.07 |  |
|  |  | Group | 0.15 | 0.07 | 4.28 | 0.045 |  |  |  |
|  | Additive | (Intercept) | 3.61 | 0.03 | 15397.43 | <0.001 |  | 0.79 | Group vs. Additive  p < 0.001 |
|  |  | ln(Duro_Pre) | 0.82 | 0.07 | 140.35 | <0.001 |  |  |  |
|  |  | Group | -0.09 | 0.04 | 5.05 | 0.030 |  |  |  |
|  | Interactive | (Intercept) | 3.64 | 0.03 | 13893.54 | <0.001 |  | 0.82 | Additive vs. Interactive p = 0.02 |
|  |  | ln(Duro_Pre) | 1.02 | 0.1 | 95.84 | <0.001 |  |  |  |
|  |  | Group | -0.11 | 0.04 | 8.36 | 0.006 |  |  |  |
|  |  | ln(Duro_Pre):Group | -0.33 | 0.13 | 6.04 | 0.018 |  |  |  |
| Skin Thickness | ln(Pre-Values) | (Intercept) | -0.17 | 0.03 | 28.68 | <0.001 |  | 0.36 | ln(Pre-Values) vs. Additive p = 0.02 |
|  |  | ln(US_Pre) | 0.65 | 0.14 | 22.72 | <0.001 |  |  |  |
|  | Group | (Intercept) | -0.1 | 0.06 | 2.78 | 0.104 |  | 0.03 |  |
|  |  | Group | -0.11 | 0.08 | 2.08 | 0.158 |  |  |  |
|  | Additive | (Intercept) | -0.08 | 0.05 | 2.8 | 0.103 |  | 0.45 | Group vs. Additive p < 0.001 |
|  |  | ln(US_Pre) | 0.69 | 0.13 | 29.23 | <0.001 |  |  |  |
|  |  | Group | -0.15 | 0.06 | 6.69 | 0.013 |  |  |  |
|  | Interactive | (Intercept) | -0.06 | 0.05 | 1.92 | 0.175 |  | 0.47 | Additive vs. Interactive p = 0.145 |
|  |  | ln(US_Pre) | 1.06 | 0.28 | 14.36 | <0.001 |  |  |  |
|  |  | Group | -0.16 | 0.06 | 7.8 | 0.008 |  |  |  |
|  |  | ln(US_Pre):Group | -0.47 | 0.31 | 2.22 | 0.145 |  |  |  |
| VT  200 Hz | ln(Pre-Values) | (Intercept) | 0.17 | 0.09 | 3.68 | 0.062 |  | 0.73 | ln(Pre-Values) vs. Additive p = 0.06 |
|  |  | ln(VPT200_Pre) | 0.94 | 0.09 | 105.36 | <0.001 |  |  |  |
|  | Group | (Intercept) | 0.31 | 0.28 | 1.25 | 0.271 |  | -0.016 |  |
|  |  | Group | -0.23 | 0.36 | 0.4 | 0.530 |  |  |  |
|  | Additive | (Intercept) | 0.38 | 0.14 | 7.81 | 0.008 |  | 0.75 | Group vs. Additive p < 0.001 |
|  |  | ln(VPT200_Pre) | 0.95 | 0.09 | 115.9 | <0.001 |  |  |  |
|  |  | Group | -0.35 | 0.18 | 3.91 | 0.055 |  |  |  |
|  | Interactive | (Intercept) | 0.37 | 0.14 | 7.53 | 0.009 |  | 0.76 | Additive vs. Interactive p = 0.22 |
|  |  | ln(VPT200_Pre) | 0.83 | 0.13 | 41.3 | <0.001 |  |  |  |
|  |  | Group | -0.35 | 0.18 | 3.88 | 0.056 |  |  |  |
|  |  | ln(VPT200_Pre):Group | 0.22 | 0.18 | 1.56 | 0.219 |  |  |  |
| VT 30 Hz | ln(Pre-Values) | (Intercept) | 2.66 | 0.04 | 3559.09 | <0.001 |  | 0.84 | ln(Pre-Values) vs. Additive p = 0.67 |
|  |  | ln(VPT30_Pre) | 0.79 | 0.06 | 200.06 | <0.001 |  |  |  |
|  | Group | (Intercept) | 2.5 | 0.17 | 209.15 | <0.001 |  | 0.01 |  |
|  |  | Group | 0.27 | 0.22 | 1.47 | 0.233 |  |  |  |
|  | Additive | (Intercept) | 2.68 | 0.07 | 1402.09 | <0.001 |  | 0.84 | Group vs. Additive p < 0.001 |
|  |  | ln(VPT30_Pre) | 0.79 | 0.06 | 186.97 | <0.001 |  |  |  |
|  |  | Group | -0.04 | 0.09 | 0.19 | 0.670 |  |  |  |
|  | Interactive | (Intercept) | 2.68 | 0.07 | 1299.87 | <0.001 |  | 0.83 | Additive vs. Interactive p = 0.96 |
|  |  | ln(VPT30_Pre) | 0.79 | 0.09 | 76.8 | <0.001 |  |  |  |
|  |  | Group | -0.04 | 0.1 | 0.18 | 0.672 |  |  |  |
|  |  | ln(VPT30_Pre):Group | -0.01 | 0.12 | 0.003 | 0.960 |  |  |  |

**Table S3:** General linear models (GLM). GLMs were used to the relationship between vibration sensitivity and the effects of skin abrasion. Pre-test values, skin hardness and skin thickness differences were set as covariates and the group variable (IG or CG) as fixed effects.

| **Dependent variable** | **Predictor** | **Coefficient** | **Standard Error** | **t-value** | **p-value** |
| --- | --- | --- | --- | --- | --- |
| **ln(skin hardness post)** | (intercept) | 1.033 | 0.311 |  |  |
|  | Group | -1.077 | 0.475 | 5.136 | 0.029 |
|  | ln(skin hardness pre) | 0.690 | 0.083 | 68.687 | <0.001 |
|  | Interaction pre*group | 0.327 | 0.133 | 6.040 | 0.019 |
| **ln(skin thickness post)** | (intercept) | -0.191 | 0.037 |  |  |
|  | Group | 0.190 | 0.063 | 9.029 | 0.005 |
|  | ln(skin thickness pre) | 0.595 | 0.140 | 18.080 | <0.001 |
|  | Interaction pre*group | 0.466 | 0.313 | 2.219 | 0.145 |
| **ln(VT 30Hz post)** | (intercept) | 0.593 | 0.241 |  |  |
|  | Group | -0.037 | 0.342 | 0.012 | 0.914 |
|  | ln(VT 30Hz pre) | 0.793 | 0.081 | 96.507 | <0.001 |
|  | ln(skin hardness Diff) | 0.009 | 0.012 | 0.580 | 0.452 |
|  | ln(skin thickness Diff) | 0.053 | 0.310 | 0.029 | 0.865 |
|  | Interaction pre*group | 0.004 | 0.124 | 0.0013 | 0.972 |
|  | (intercept) | -0.288 | 0.399 |  |  |
|  | Group | 0.215 | 0.237 | 1.606 | 0.215 |
| **ln(VT 200Hz post)** | ln(VT 200Hz pre) | 1.073 | 0.286 | 65.253 | <0.001 |
|  | ln(skin hardness Diff) | 1.124 | 0.964 | 0.748 | 0.394 |
|  | ln(skin thickness Diff) | -0.491 | 0.539 | 0.164 | 0.688 |
|  | Interaction pre*group | -0.81 | 0.189 | 1.453 | 0.237 |

**Table S4:** Comparative plantar hardness values.

| **Article** | **Population** | **Age [yrs]** | **n** | **Measurement spot** | **Hardness mean**  **(± dispersion) [Sh]** |
| --- | --- | --- | --- | --- | --- |
|  |  |  |  |  |  |
|  |  |  |  |  |  |
| Strzalkowski  et al. 2015 | healthy | 24 | 22 | Hallux  Met 5  Med. Arch  Lat. Arch  Heel | 40  43  34  41  46 |
|  |  |  |  |  |  |
|  |  |  |  |  |  |
| Jammes  et al. 2017 | healthy | 23 | 15 | Met 1 & 2  Met 5  Heel | 30 ± 4  60 ± 3  50 ± 2 |
|  | healthy | - | 6 | Met 5 | 70 ± 3 |
|  |  |  |  |  |  |
|  |  |  |  |  |  |
| Holowka  et al. 2019 | healthy | 35 | 46 | Met 1  Heel | 31 ± 9  43 ± 10 |
|  | healthy barefoot | 41 | 35 | Met 1  Heel | 40 ± 11  54 ± 12 |
|  |  |  |  |  |  |
|  |  |  |  |  |  |
| Helili  et al. 2021 | healthy | 44 | 59 | Med. Forefoot  Lat. Forefoot  Lat. Midfoot  Med. Rearfoot  Lat. Rearfoot | 29 ± 4  30 ± 5  28 ± 6  34 ± 7  34 ± 7 |
|  |  |  |  |  |  |
|  |  |  |  |  |  |
| Zippenfennig  et al. 2021 | healthy | 56 | 33 | Met 1  Heel | 29 ± 14  29 ± 8 |
|  | diabetic | 53 | 20 | Met 1  Heel | 27 ± 8  33 ± 8 |
|  | neuropathic | 61 | 13 | Met 1  Heel | 31 ± 8  39 ± 9 |
|  |  |  |  |  |  |
|  |  |  |  |  |  |
| Allan  et al. 2022 | diabetic | 63 | 39 | Hallux  Met 1  Met 3  Met 5  Midfoot  Heel | 38  39  35  33  39  43 |
